# Adherence to stewardship recommendations for antibiotic discontinuation reduces antibiotic-associated adverse drug events

**DOI:** 10.1017/ash.2024.29

**Published:** 2024-03-18

**Authors:** Patrick Mulligan, Nader Ismail, Nirav Shah, Jessica P. Ridgway, Urmila Ravichandran, Jennifer Grant, Mary Ellen Acree

**Affiliations:** 1 NorthShore University HealthSystem, Evanston, IL, USA; 2 University of Chicago Pritzker School of Medicine, Chicago, IL, USA

## Abstract

Inappropriate antibiotic use may lead to increased adverse drug events (ADEs). This study assessed whether an antimicrobial stewardship recommendation to discontinue antibiotics in patients with low likelihood for bacterial infection reduced antibiotic duration and antibiotic-associated ADEs. At a 4-hospital system, hospitalized adult patients receiving empiric antibiotics for suspected infection were identified between May 2, 2016 and June 30, 2018. For those patients who were deemed unlikely to have a bacterial infection, a note was left by an infectious diseases physician recommending antibiotic discontinuation. Patient cases were considered “adherent” to recommendations if antibiotics were discontinued within 48 hours of the note and “non-adherent” to recommendations if antibiotics were continued beyond this. Duration of antibiotics and potential antibiotic-associated ADEs were collected retrospectively. Attribution of the adverse event to the antibiotic was decided upon by the investigators. The incidence of ADEs and duration of antibiotics between the adherent and non-adherent groups were compared. Of 253 patients deemed unlikely to have a bacterial infection, 114 (45%) treatment teams stopped antibiotics within 48 hours of the recommendation, and 139 (55%) continued antibiotics. The total number of ADEs was significantly higher in the non-adherent group compared to the adherent group (34 ADEs vs 9 ADEs, *P* = .001). The median number of total prescribed antibiotic days was higher in the non-adherent group than in the adherent group (5 days vs 1 day, *P* < .001). This study demonstrates that stewardship programs may prevent ADEs by recommending antibiotic discontinuation in patients with low suspicion for bacterial infection.

## Introduction

Inappropriate antibiotic use leads to deleterious consequences including increased antibiotic resistance, healthcare costs, and rates of adverse drug events (ADEs). ADEs are side effects from drug therapy, ranging from gastrointestinal upset to severe organ toxicity. In a study by Tamma et al.,^1^ ADEs occurred in ∼20% of inpatients receiving non-clinically indicated antibiotics. In this study, we assessed whether an antimicrobial stewardship recommendation to discontinue antibiotics in patients with a low likelihood for bacterial infection reduced antibiotic duration and antibiotic-associated ADEs.

## Methods

At NorthShore University HealthSystem, a 4-hospital, 832-bed system based in the Chicago suburbs, hospitalized patients aged ≥18 years receiving empiric antibiotics for suspected infection were identified through the electronic medical record (EMR) between May 2, 2016 and June 30, 2018. Patients were excluded if they had an unclear focus of infection, multiple sources of infection, were receiving pathogen-directed therapy (rather than empiric therapy), or had an infectious disease (ID) consultation. 7 days per week, an ID physician reviewed the patient’s chart within 24 hours of antibiotic initiation. For those patients who were deemed unlikely to have a bacterial infection, a note was left in the EMR recommending antibiotic discontinuation, and the treating team was paged. Most often these patients were believed to have asymptomatic bacteriuria or a viral illness. Patient cases were considered “adherent” to ID recommendations if antibiotics were discontinued within 48 hours of the note and “non-adherent” to recommendations if antibiotics were continued beyond this.

Two investigators (P.M. and N.I.) performed a retrospective chart review, collecting data on demographics, duration of antibiotics, and potential antibiotic-associated ADEs within 30 days following the recommendation to discontinue antibiotics. ADE definitions (Supplement) were derived from the study by Tamma et al.,^1^ and the adverse event must be known to occur with at least 1 antibiotic used for that patient. Evidence of the ADE had to be documented clearly in nursing notes, progress notes, and/or office visit notes or had to be documented in the results tab for groups with strict lab value changes such as the hematologic, hepatobiliary, renal, cardiac, or musculoskeletal ADEs. Attribution of the adverse event to the antibiotic was decided upon by the investigators based on chart review. Disagreement between investigators on the presence of an ADE prompted a tie-breaker review (J.G. or M.A.). The investigators performing chart review were not blinded to group assignment (adherent vs non-adherent) nor were the tie-breaker reviewers. We compared the incidence of ADEs by individual categories and composite score as well as duration of total antibiotic therapy between the adherent and non-adherent groups. For patients with more than one ADE, only one ADE contributed to the composite score. Continuous variables were compared using Wilcoxon rank sum test and represented as median (interquartile range) if there was a skewed distribution or compared using *t* test and represented as mean (standard deviation). Indicator variables were compared using χ^2^ test or Fisher's exact test where applicable. Statistical significance of the composite ADE score and duration of antibiotic therapy was calculated using Bonferroni adjusted alpha level of 0.0055 (0.05/9) to account for multiple comparisons.

## Results

253 patients were identified as unlikely to have a bacterial infection, and a recommendation advising antibiotic discontinuation was given. Of these 253 cases, 114 (45%) treatment teams stopped antibiotics within 48 hours of the recommendation, and 139 (55%) continued antibiotics beyond this period. The baseline characteristics of the two groups, including age, gender, race/ethnicity, and Acute Physiology and Chronic Health Evaluation (APACHE II) score, were evaluated, and no statistically significant differences were observed. Overall, the most common ADEs were hematologic and nausea. There were no anaphylactic, hepatobiliary, cardiac, or musculoskeletal ADEs identified. The total number of ADEs was significantly higher in the non-adherent group compared to the adherent group (34 ADEs vs 9 ADEs, *P* = .001) (Table 1). No individual ADE category met the threshold for statistical significance, though the nausea and non-*Clostridioides difficile* diarrhea categories were closest to achieving this threshold. The median number of total prescribed antibiotic days was higher in the non-adherent group than in the adherent group (5 days vs 1 day, *P* < .001). There were no statistically significant differences between the adherent and non-adherent groups for the outcomes of 30-day readmission (13% vs 22%, *P* = .114) or 30-day mortality (5% vs 3%, *P* = .519).

**Table 1.** Antibiotic-Associated Adverse Drug Events and Antibiotic Duration in Adherent and Non-Adherent Groups

	Overall 253	Recommendation followed 114 (45%)	Recommendation not followed 139 (55%)	*P* value
**ADE attributable to antibiotic**				
Composite ADE^a^	43 (17.00)	9 (7.89)	34 (24.46)	**.001** ^b^
* C. difficile*	9 (3.56)	3 (2.63)	6 (4.32)	.705
Dermatologic	2 (0.79)	0 (0.00)	2 (1.44)	.567
Hematologic	21 (8.30)	6 (5.26)	15 (10.79)	.175
Nausea	10 (3.95)	1 (0.88)	9 (6.47)	.051
Neurologic	1 (0.40)	0 (0.00)	1 (0.72)	1.000
Non-*C. difficile* diarrhea	6 (2.37)	0 (0.00)	6 (4.32)	.067
Renal	3 (1.19)	0 (0.00)	3 (2.16)	.320
**Other outcomes**				
Length of total treatment, days	3.00 (1.00, 6.00)	1.00 (1.00, 2.00)	5.00 (4.00, 7.00)	**<.001** ^b^

Note. ADEs, adverse drug events.

There were no anaphylactic, hepatobiliary, cardiac, or musculoskeletal ADEs identified.

a Composite ADE: only one ADE per patient included.

b Statistically significant with Bonferroni-adjusted alpha level of 0.0055 (0.05/9) to account for multiple comparisons.

## Discussion

Our study demonstrates that an antimicrobial stewardship intervention can decrease antibiotic days of therapy and ADEs when antibiotics are not indicated. This study is limited by its small size and retrospective nature. A lack of blinding of the individuals performing chart review may have contributed to bias when determining attribution of the ADE to antibiotic exposure. However, the investigators were trained on the use of strict criteria for each of the ADE categories with few patient cases requiring tie-breaker review. Additionally, other than an evaluation of 30-day readmission and 30-day mortality, we did not assess if there were any harms from withholding antibiotics for the patients for whom the recommendation was followed. We also did not collect data on why recommendations were or were not followed. A strength of this study includes the detailed chart review for ADEs using previously established criteria. The findings of this study support previous research showing the impact of antimicrobial stewardship on reduction in antibiotic-associated ADEs.^2,3^ While prior research has focused on the prevention of ADEs through shortened duration of antibiotic courses, particularly at care transitions, this study shows that stewardship programs may prevent ADEs by recommending antibiotic discontinuation in patients with low suspicion for bacterial infection.

## Supporting information

Mulligan et al. supplementary material Mulligan et al. supplementary material

## Supplementary material

For supplementary material accompanying this paper visit http://doi.org/10.1017/ash.2024.29. click here to view supplementary material
