# Supplementary material for: Adherence to stewardship recommendations for antibiotic discontinuation reduces antibiotic-associated adverse drug events
Source: Antimicrob Steward Healthc Epidemiol. 2024 Mar 18;4(1):e36. doi: 10.1017/ash.2024.29 (PMC10945931; doi:10.1017/ash.2024.29)
Supplement: Mulligan et al. supplementary material [file S2732494X24000299sup001.docx]

**Supplemental Material**

| **ADE Definition Exceptions** | | |
| --- | --- | --- |
| *C. difficile* | *C. difficile* positive PCR and diarrhea | Laxative use |
| Dermatologic | Rash, including hives, non-hives rashes | Rash prior to antibiotic use, other documented suspected explanation |
| Hematologic | One or more lab values of: Hgb <10mg/dL, WBC < 4,500 cells/µL or platelets <150,000 cells/µL | Lab values must be below prior established baseline and no pre-existing myelosuppressive illness or documentation of bleeding |
| Nausea | Nausea or emesis | Alternative explanation provided in EMR or recorded use of oral contrast or tube feeds |
| Neurologic | Altered mental status, peripheral neuropathy or seizure activity | Symptoms present prior to admission, use of non-antibiotic medications that could lower seizure threshold, use of other neurologically impactful medications/substances |
| Non *C. difficile* Diarrhea | >3 loose stools per day, documentation of diarrhea | *C. difficile* diagnosis, stool softeners, tube feeding, oral contrast, gastroenteritis |
| Renal | Serum creatinine >1.5 times patient’s baseline creatinine prior to antibiotic usage | Shock, IV contrast use, common nephrotoxin use prior to elevation of creatinine |
| Anaphylactic | Acute onset respiratory compromise, hypotension or end-organ dysfunction reported within minutes of antibiotic administration | Other allergen exposure |
| Hepatobiliary | One or more lab values of: total bilirubin >3mg/dL, AST >120 U/L, ALT >189 U/L | Pre-existing hepatobiliary disease or recent biliary instrumentation |
| Cardiac | QTc in two or more ECGs >440 ms in males and >460 ms in females | Previous ECGs with prolonged QTc or history of arrhythmia |
| Musculoskeletal | Serum creatine kinase >1000 U/L | Previous history of myopathy, known crush injury or compartment syndrome, statin use |
